# Psychometric and structural properties of Traditional Chinese version of postoperative oral dysfunction-10 in patients with oral cancer undergoing facial free flap reconstruction surgery

**DOI:** 10.1186/s12885-026-16470-9

**Published:** 2026-07-06

**Authors:** Wen-Chuan Hsu, Wen-Pin Yu, Hui-Mei Han, Huang-Kai Kao, Hsiao-Yean Chiu

**Affiliations:** 1 School of Nursing, College of Nursing, Taipei Medical University, 250 Wu-Hsing St., Taipei, 110 Taiwan; 2 Department of Nursing, Chang Gung Memorial Hospital, Linkou, Taiwan; 3 Chang Gung University of Science and Technology, Taoyuan, Taiwan; 4 Department of Plastic and Reconstructive Surgery, Chang Gung Memorial Hospital, Linkou, Taiwan; 5 Department of Plastic and Reconstructive Surgery, Chang Gung University, Taoyuan, Taiwan; 6 Department of Nursing, Taipei Medical University Hospital, Taipei, Taiwan

**Keywords:** Dysphagia, Free-flap reconstruction, Reliability, Validity, Oral cancer

## Abstract

**Purpose:**

Dysphagia is a frequent complication in oral cancer patients after free-flap reconstruction. Although the Postoperative Oral Dysfunction-10 (POD-10) is widely used to assess swallowing dysfunction, no validated Traditional Chinese version exists for Mandarin-speaking Taiwanese patients. This study aimed to translate the POD-10 into Traditional Chinese (TC-POD-10) and evaluate its reliability and validity in Mandarin-speaking Taiwanese patients with oral cancer undergoing free-flap reconstruction.

**Methods:**

A cross-sectional study was conducted with 100 oral cancer patients post free-flap surgery in a medical center. Internal consistency was tested using Cronbach’s alpha. Construct validity was evaluated through exploratory factor analysis (EFA). Criterion validity was assessed by correlating TC-POD-10 with the Chinese Functional Oral Intake Scale (FOIS). Known-group validity was tested using Eating Assessment Tool-10 (EAT-10) scores to distinguish between patients with and without dysphagia. Diagnostic accuracy was determined using the area under the receiver operating characteristic curve (AUC).

**Results:**

The TC-POD-10 demonstrated excellent internal consistency (α = 0.94) and strong correlation with FOIS (*r* = -0.89, *p* < 0.001). Patients with dysphagia had significantly higher TC-POD-10 scores (14.6 vs. 10.0, *p* < 0.001). EFA revealed a single-factor model explaining 65.6% of variance. The tool showed high accuracy (AUC = 0.92), with sensitivity of 89% and specificity of 96% at a cutoff score of 15.

**Conclusion:**

The TC-POD-10 is a reliable and valid instrument for assessing dysphagia in Taiwanese oral cancer patients. Its strong psychometric performance supports its clinical use for evaluating and managing swallowing dysfunction.

## Introduction

Dysphagia is highly prevalent among patients with oral cancer undergoing free-flap reconstruction, and its incidence rate ranges from 41.3% to 88.0% and reaches 98.0% during the first postoperative week [1–3]. Although free-flap reconstruction surgery effectively restores oral structures, it may damage muscles, nerves, and organs, impairing sensory and motor functions essential for swallowing [4–6]. Thus, patients may experience eating difficulties, malnutrition, dehydration, and aspiration pneumonia, which severely affect health and recovery [7, 8]. Structural changes resulting from reconstructive surgery may also cause anxiety and fear, negatively affecting daily life, social interaction, and overall quality of life [1].

Traditionally, objective swallowing assessment tools, including the video fluoroscopic swallowing study (VFSS) [9, 10] and the flexible endoscopic examination of swallowing (FEES) [11], are considered the gold standard for diagnosing dysphagia. However, these tools require specialized personnel and equipment, which makes them less accessible [9, 12]. Moreover, the clinical implementation of these methods is costly, time-consuming, and difficult [13]. Recently, the water swallow test (WST) was reported to be relatively robust for patients with head and neck cancer (HNC), demonstrating a pooled sensitivity of 0.82 and a pooled specificity of 0.79 [14].

Self-administered screening questionnaires provide a simple, low-cost, and efficient method for the diagnosis of dysphagia. Commonly used instruments include the M.D. Anderson Dysphagia Inventory (MDADI) [15], Eating Assessment Tool-10 (EAT-10) [16], and Functional Oral Intake Scale (FOIS) [17]. The MDADI is specifically designed for patients with HNC to investigate the effect of dysphagia on their quality of life. The EAT-10, a 10-item self-assessment scale, is a simple and effective tool for evaluating the physiological, psychological, and social effects of dysphagia in various populations, including patients with stroke, HNC, and gastrointestinal conditions [18]. The FOIS primarily evaluates the oral intake ability of patients with stroke by categorizing their functional eating status. Although the FOIS provides information on the oral intake level, its applicability and validity in other patient populations require further investigation. Although these instruments offer valuable insights, their applicability to patients with oral cancer undergoing free-flap reconstruction remains limited because of differences in dysphagia etiology and manifestation in this population, such as impaired masticatory function, increased food residue in the oral cavity, sialorrhea, organic articulation disorders, and negative effects on social and psychological well-being [3, 19–22].

The Postoperative Oral Dysfunction-10 (POD-10) has been established as a clinically reliable and effective tool for assessing postoperative oral dysfunction in patients with oral cancer [4, 23]. Given the substantial effect of dysphagia on postoperative recovery, the POD-10 serves as an essential instrument for evaluating swallowing difficulties. By examining patient-reported swallowing function, the POD-10 facilitates the identification of dysphagia severity and guides appropriate interventions to improve postoperative outcomes.

The present study translated the POD-10 into Traditional Chinese (TC-POD-10) for patients with oral cancer undergoing free-flap reconstruction surgery and determined its reliability and validity. The TC-POD-10 can thus be used to develop individualized swallowing care plans, monitor changes in swallowing function on a daily basis, and identify secondary oral complications following surgery.

## Methods

This study comprised a translation phase and psychometric testing phase. In the first phase, the Japanese version of the POD-10 was translated into the TC-POD-10. In the second phase, the TC-POD-10 was subjected to psychometric testing.

### Translation process

Permission was first obtained from the developer of the POD-10, Dr. Matsuda, to translate the instrument into Traditional Chinese. The instrument translated in this study was the Postoperative Oral Dysfunction-10 (POD-10), developed by Matsuda et al. (2021). The name was retained in its original form to ensure conceptual and terminological consistency with the source version. The translation was conducted in accordance with established guidelines [24]. Three translators who were native Mandarin Chinese speakers proficient in Japanese independently translated the Japanese version of the POD-10 into Traditional Chinese. The three translated versions were then discussed, compared, and merged into a single, complete version. Subsequently, two back-translators who were native Japanese speakers proficient in Chinese independently translated the Traditional Chinese version back into Japanese. A panel discussion involving a nursing expert, an English lecturer, and two nurse practitioners was conducted to analyze and discuss the back-translated version. After these steps were completed, the TC-POD-10 was finalized.

### Psychometric testing

#### Study design and settings

A cross-sectional research design with consecutive sampling was used for conducting the second-phase validation for reducing selection bias. This study was conducted in the plastic surgery ward of a medical center in northern Taiwan from June 2024 to March 2025. This study was approved by the Institutional Review Board of Chang Gung Memorial Hospital (IRB No. 202400312B0) and conducted in accordance with the principles of the Declaration of Helsinki. A trained research nurse obtained written informed consent from each participant after providing verbal and written explanations of the study purpose, procedures, and rights. Participation was voluntary, and participants were informed that they could withdraw at any time without any consequence to their medical treatment. All collected data were anonymized and securely stored to ensure confidentiality.

#### Study population

The inclusion criteria were being aged 18 years or older, having received a diagnosis of oral cancer, and having undergone facial free-flap reconstruction surgery. All participants were admitted to the plastic surgery ward on postoperative day 1. The exclusion criteria were being unable to read or write Traditional Chinese and having preexisting dysphagia before the cancer diagnosis.

The minimum required sample size was estimated using a subject-to-item ratio, following the widely accepted standard of 10 participants per measurement item, which is considered adequate for exploratory factor analysis and psychometric testing [25, 26]. For the 10-item POD-10 scale, a sample size of 100 participants was therefore required.

#### Instruments

A pre-designed information form was used to collect data on demographic and disease characteristics associated with the risk of dysphagia in patients with oral cancer who underwent flap reconstruction [4, 7]. The variables were age, sex, education level, body mass index, smoking status, alcohol consumption, betel quid chewing status, tumor grade, tumor site, flap reconstruction site, neck lymph node clearance, and radiotherapy and chemotherapy status. To determine the effects of dysphagia, additional variables such as nasogastric tube placement and tracheostomy before discharge were retrieved from patients’ electronic health records.

The POD-10 developed by Matsuda et al. (2021) is a self-reported questionnaire designed to subjectively assess oral function following oral cancer treatment [4]. The instrument comprises10 items, each rated on a 5-point Likert scale (0 = *no problem*; 4 = *severe problem*), for a total score ranging from 0 to 40. Higher scores indicate more impaired oral function. A cutoff score of ≥ 24 points was established relative to two clinical indicators: the FOIS (corresponding to grades 6 and 7 vs. grades 1–5) and the Mini Nutritional Assessment–Short Form (corresponding to normal nutritional status vs. at risk of malnutrition) [4].

To assess criterion validity, the FOIS, a 7-point ordinal scale for evaluating the level of oral intake in patients with dysphagia, was used [17]. Higher FOIS scores indicate better swallowing function, ranging from total reliance on tube feeding (Level 1) to normal oral intake without restrictions (Level 7) [27]. The FOIS had high internal consistency (Cronbach’s alpha = 0.9) and acceptable validity, correlating with other functional assessment tools [17]. The FOIS is widely used in clinical and research settings to evaluate swallowing function, monitor recovery progress, and guide dietary modifications for patients with dysphagia, particularly those recovering from stroke or oral cancer treatments. The Chinese version of the FOIS was validated for patients with HNC [28]. The results revealed high interrater consistency (k = 0.790, *p* < 0.001). The Chinese version of the FOIS exhibited a moderate negative correlation with the Chinese version of the EAT-10 (*r* = − 0.533, *p* < 0.001), indicating good reliability and validity [28].

To evaluate known-group validity, the EAT-10, a 10-item, self-administered questionnaire designed to assess dysphagia severity, was used. Each item is scored on a 5-point Likert scale (0 = *no problem*; 4 = *severe problem*), with total scores ranging from 0 to 40. Higher scores indicate greater swallowing difficulty. The EAT-10 demonstrated high internal consistency (Cronbach’s alpha = 0.960) and acceptable test–retest reliability (*r* = 0.72–0.91). A score of 3 or higher is considered abnormal, making the EAT-10 a reliable tool for screening dysphagia severity and monitoring treatment outcomes in various swallowing disorders [16]. The Chinese version of the EAT-10, validated for individuals with swallowing disorders, had high interrater reliability (k = 0.919) and strong validation outcomes [18].

The bedside 3-ounce water swallowing test was administered to objectively screen for postoperative dysphagia. Each participant was instructed to drink 3 ounces of water from a cup in one continuous swallow while seated upright. Clinical signs such as coughing, choking, throat clearing, or wet/hoarse voice were observed during and immediately after swallowing. The presence of any of these signs was considered indicative of swallowing impairment. The 3-oz WST is a simple, non-invasive, and validated bedside screening tool with high diagnostic accuracy, demonstrating pooled sensitivity of 0.82 and specificity of 0.79 for detecting dysphagia in head and neck cancer populations [14]. Earlier studies have reported even higher sensitivity (96.5%) for detecting aspiration risk [29]. The test was performed by a speech therapist and an attending physician within 7 days postoperatively to confirm the presence or absence of dysphagia.

#### Procedures

A research nurse reviewed the participant list daily to identify eligible patients. After providing their written informed consent, the participants underwent a comprehensive dysphagia assessment on the seventh postoperative day following flap reconstruction for oral cancer. If a participant was discharged earlier than expected, all assessments were completed on the day of discharge. During the research period, the research nurse examined patients by using the POD-10, and all participants underwent a 3-ounce water swallowing test to evaluate dysphagia. A speech therapist and specialist physician performed the water swallowing test for diagnostic confirmation. To minimize assessment bias, the POD-10 assessor was blinded to the diagnostic results of the WST, and the WST evaluators were blinded to the POD-10 scores. To establish criterion and known-group validity, evaluations were conducted using the FOIS, EAT-10, and POD-10 questionnaires, each requiring approximately 5 min to complete.

#### Statistical analysis

All analyses were performed using IBM SPSS. Statistics for Windows version 20.0 (IBM, Armonk, New York, USA), and a P value of < 0.05 was regarded as statistically significant.

### Descriptive analysis

Descriptive analyses were performed to examine the demographic and disease characteristics and questionnaire scores of the participants. To examine the distribution of potential confounders between patients with and without dysphagia, we conducted between-group comparisons. Continuous variables were compared with independent-samples t tests, and categorical variables with chi-square tests or Fisher’s exact tests, as appropriate.

### Validity

Exploratory factor analysis (EFA) with orthogonal rotation was conducted to examine construct validity. In addition, the Kaiser–Meyer–Olkin (KMO) test and Bartlett’s test of sphericity were performed to evaluate the correlation matrix and the adequacy of the sample. The KMO index ranges from 0 to 1, with values greater than 0.70 considered acceptable. A *p* value of less than 0.05 in Bartlett’s test of sphericity indicates statistical significance.

To assess criterion validity, Pearson’s correlation coefficients were used to examine the relationship between the TC-POD-10 and FOIS. Known-group validity was evaluated using independent samples *t*-tests to compare TC-POD-10 total scores between individuals with and without dysphagia, as defined by EAT-10 scores of < 3 and ≥ 3, respectively.

Receiver operating characteristic (ROC) analysis was performed to determine the optimal cutoff score of the POD-10 by using the WST as the reference standard. The WST is the routine dysphagia assessment at the study hospital and is conducted by a speech therapist and a specialist physician on the basis of established criteria. The area under the ROC curve (AUC) for the TC-POD-10 was calculated to distinguish participants with dysphagia from those without dysphagia. A higher AUC value indicates more accurate prediction, and a value of ≥ 0.8 is considered favorable for discrimination [30]. Youden’s index (J = sensitivity + specificity − 1) was adopted to determine the optimal cutoff point [31].

### Reliability

The Cronbach’s alpha for each item and the item-total correlations of the TC-POD-10 were calculated using Pearson’s product-moment correlation coefficients. A Cronbach’s alpha greater than 0.70 was considered indicative of adequate internal consistency. Test–retest reliability was evaluated using intraclass correlation coefficient (ICC).

## Results

### Distribution of demographic and disease characteristics

The study included 100 participants who were eligible for admission after undergoing flap reconstruction surgery for oral cancer. Among these participants, 52 (52.0%) experienced dysphagia per the WST, whereas 48 (48.0%) did not. The baseline characteristics of the participants are summarized in Table 1. The distribution of patients with and without dysphagia and differences in their demographic characteristics and treatment-related variables were determined. Significant differences were observed between the two groups regarding the use of a nasogastric tube and diet type before discharge. Subgroup analyses by tumor site, reconstruction flap type, and prior adjuvant therapy showed no statistically significant differences in TC-POD-10 scores (Supplementary Table S1).

**Table 1:** Baseline characteristics of study participants

	Total(*n* = 100)		With dysphagia(*n* = 52)		Without dysphagia(*n* = 48)		*P* value
Characteristics	*n*	*(%)*	*n*	(%)	*n*	(%)	
Age, mean (SD)^a^	58.6	(10.4)	59.5	(10.2)	57.7	(10.5)	0.62
Sex^b^							0.88
Male	88	(88.0)	46	(88.5)	42	(87.5)	
Female	12	(12.0)	6	(11.5)	6	(12.5)	
Tobacco exposure^b^	80	(80.0)	43	(82.7)	37	(77.1)	0.48
Alcohol consumption^b^	76	(76.0)	40	(76.9)	36	(75.0)	0.82
Betel nut^b^	82	(82.0)	44	(84.6)	38	(79.2)	0.48
Tumor site^c^							0.24
Buccal cancer	37	(37.0)	19	(36.5)	18	(37.5)	
Tongue cancer	14	(14.0)	7	(13.5)	7	(14.6)	
Gum tumor	32	(32.0)	20	(38.5)	12	(25.0)	
Pharyngeal	6	(6.0)	4	(7.7)	2	(4.2)	
Jaw	6	(6.0)	1	(1.9)	5	(10.4)	
Mouth floor	5	(5.0)	1	(1.9)	4	(8.3)	
Pathological results^c^							0.09
Squamous cell	93	(93.0)	51	(98.1)	42	(87.5)	
Adenocarcinoma	5	(5.0)	1	(1.9)	4	(8.3)	
Sarcoma	2	(2.0)	0	(0.0)	2	(4.2)	
Tumor stage^c^							0.45
0	2	(2.0)	0	(0.0)	2	(4.2)	
I	6	(6.0)	4	(7.7)	2	(4.2)	
II	20	(20.0)	9	(17.3)	11	(22.9)	
III	14	(14.0)	6	(11.5)	8	(16.7)	
IV	58	(58.0)	33	(63.5)	25	(52.1)	
Node stage^c^							0.75
0	48	(48.0)	22	(42.3)	26	(54.2)	
I	3	(3.0)	2	(3.9)	1	(2.1)	
II	41	(41.0)	23	(44.2)	18	(37.5)	
III	7	(7.0)	4	(7.7)	3	(6.3)	
IV	1	(1.0)	1	(1.9)	0	(0.0)	
Neck dissection^b^	86	(86.0)	42	(80.8)	44	(91.7)	0.39
Types of reconstruction flap^c^							0.14
Profunda artery perforator flap	9	(9.0)	3	(5.8)	6	(12.5)	
Fibula	39	(39.0)	23	(44.2)	16	(33.3)	
Anterolateral Thigh flap	41	(41.0)	19	(36.5)	22	(45.8)	
Deltoid	2	(2.0)	2	(3.9)	0	(0.0)	
Latissimus Dorsi	2	(2.0)	0	(0.0)	2	(4.2)	
Groin flap	1	(1.0)	0	(0.0)	1	(2.1)	
Profunda artery perforator flap + fibula	2	(2.0)	1	(1.9)	1	(2.1)	
Profunda artery perforator flap + Anterolateral Thigh flap	1	(1.0)	1	(1.9)	0	(0.0)	
Fibula + Anterolateral Thigh flap	3	(3.0)	3	(5.8)	0	(0.0)	
Preoperative radiotherapy^b^	42	(42.0)	25	(48.1)	17	(35.4)	0.20
Preoperative chemotherapy^b^	48	(48.0)	30	(57.7)	18	(37.5)	0.07
Tracheotomy after treatment^b^	41	(41.0)	20	(38.5)	21	(43.8)	0.59
NG tube placement before discharge^b^	78	(78.0)	45	(86.5)	33	(68.8)	0.03
Diet types before discharge^c^							0.03
Liquid	81	(81.0)	47	(90.4)	34	(70.8)	
Soft	8	(8.0)	3	(5.8)	5	(10.4)	
Full	11	(11.0)	2	(3.9)	9	(18.8)	

*Abbreviation*: *SD* Standard deviation, *NG* Nasogastric tube

^a^Independent t-test

^b^Chi squared test

^c^Fisher’s exact test

### Construct validity

The EFA findings are presented in Table 2. The results indicated satisfactory sampling adequacy and confirmed that the correlation matrix was not an identity matrix (KMO = 0.945, *p* < 0.001). Each item had an acceptable factor loading (> 0.7). A one-component structure, specifically focused on dysphagia, was identified as the best-fitting model, explaining 65.6% of the total variance.

**Table 2:** Factor extractions of exploratory factor analysis

No	Items (Japanese/English/Traditional Chinese)	Mean ± SD	Factor loading	Eigenvalue	Variance (%)
1	お口の問題が原因で、食べるのに時間がかかる It takes a long time to eat because of mouth problems. 因為口腔問題, 您花更多時間在吃東西上	1.25 ± 1.12	0.82		
2	お口の問題が原因で、食べ物に制限がある There are food restrictions due to mouth problems. 因為口腔問題, 您選擇吃的食物受到限制	1.47 ± 1.13	0.80		
3	お口の問題が外食に行くための障害になっている Mouth problems have become a barrier to going out to eat. 您口腔的問題造成無法外出用餐	1.18 ± 1.11	0.85		
4	固形物を噛む時に、余分な努力が必要だ It takes extra effort to chew solid food. 當您咀嚼固體食物時, 需要更費力	1.69 ± 1.09	0.70		
5	お口に食べ物が残ってしまう Food will remain in your mouth. 進食時, 食物會殘留在您的口腔	1.61 ± 0.97	0.77		
6	お口から食べ物や唾液がこぼれる Food or saliva spills from your mouth. 食物或口水會從口腔流出來	1.50 ± 1.21	0.82		
7	お口が乾く I feel my mouth getting dry. 口腔會感到乾	1.48 ± 1.11	0.85		
8	しゃべることが苦痛だ I find it painful to talk 說話時口腔會感覺疼痛	1.71 ± 1.11	0.79		
9	お口の問題が原因で、相手に言葉が伝わらない Due to mouth problems, the other person cannot understand your words. 因為口腔的問題, 無法用言語將訊息傳達給對方	1.39 ± 1.11	0.84		
10	お口の見た目に自信がない I am not confident about the appearance of my mouth. 口腔的外觀使您沒有自信	1.28 ± 0.99	0.83		
Total variance (%)				6.56	65.6
Kaiser-Meyer-Olkin (Bartlett’s Test of Sphericity)		0.945 (*p* < 0.001)			

*AbbreviationsM* mean, *SD* Standard deviation

### Criterion validity

The participants’ total and item scores on the TC-POD-10 were significantly and negatively correlated with their total FOIS scores (*r* = -0.89, all *p* < 0.001), indicating that the TC-POD-10 effectively assesses dysphagia following flap reconstruction for oral cancer.

### Known-group validity

Independent samples *t*-tests were conducted to examine differences in TC-POD-10 total scores between patients with and without dysphagia, as distinguished by the EAT-10. A significant difference in TC-POD-10 total scores was observed between the dysphagia (EAT-10 score ≥ 3) and nondysphagia (EAT-10 < 3) groups (mean = 10.0 vs. 14.6, *p* < 0.001).

### ROC analysis

**Fig. 1:**
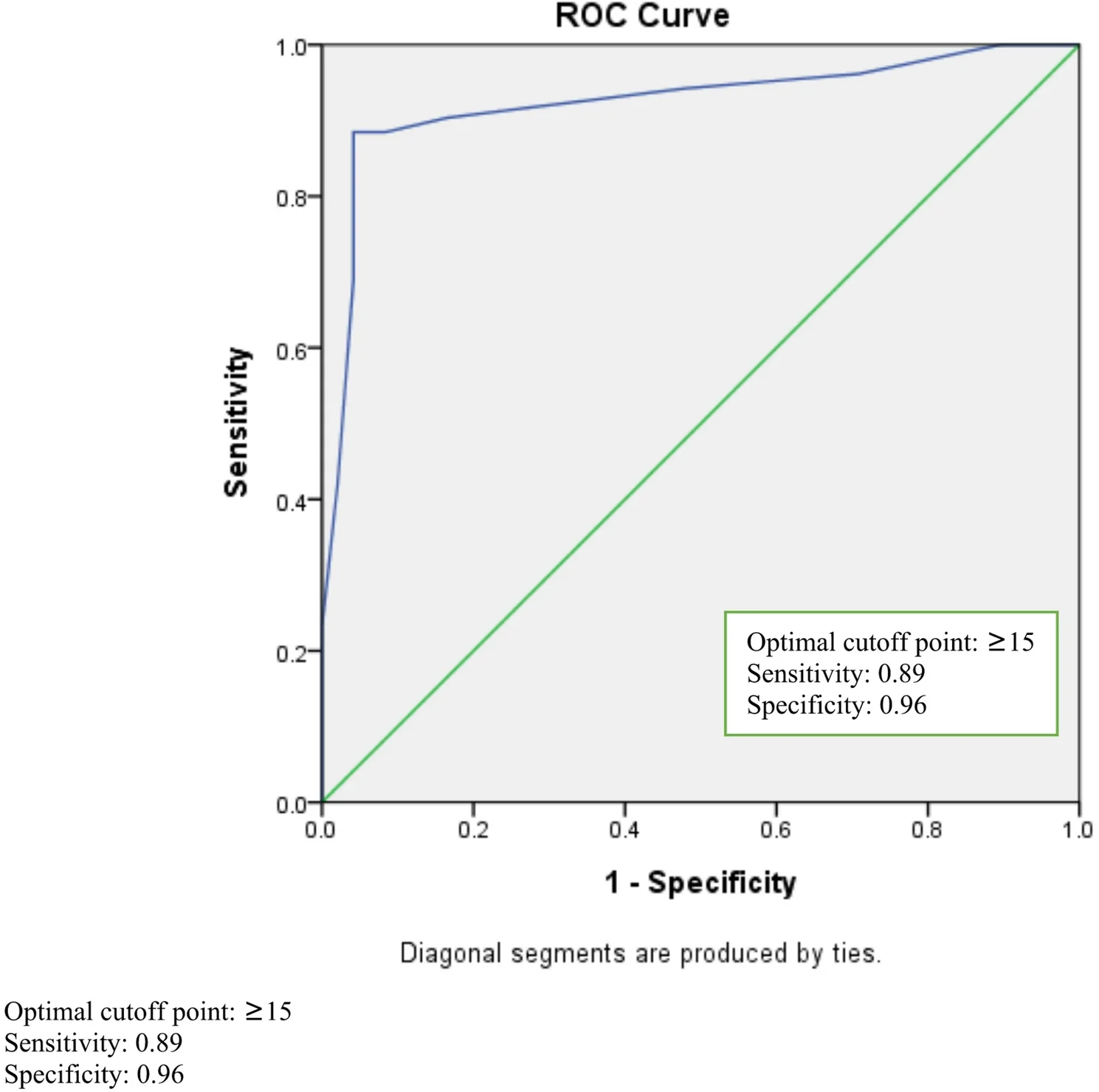
Receiver operating characteristic curve of the Traditional Chinese version of the Postoperative Oral Dysfunction-10 for detecting dysphagia, using the Water Swallow Test and clinical evaluation as the reference standard (*n* = 100). The optimal cutoff score of 15 is shown (AUC = 0.92, 95% CI: 0.87–0.98; sensitivity = 0.89; specificity = 0.96)

The results of the ROC analysis are presented in Fig. 1. When benchmarked against the WST, TC-POD-10 total score was highly predictive of dysphagia, with a sensitivity of 89% and a specificity of 96%. The optimal cutoff was determined to be 15 on the basis of the WST and a dysphagia evaluation conducted by a speech therapist and a specialist physician. The AUC for the TC-POD-10 was 0.92 (95% CI, 0.87–0.98), indicating excellent diagnostic accuracy.

### Reliability

The TC-POD-10 demonstrated high reliability, with a Cronbach’s alpha coefficient of 0.94. Item-level Cronbach’s alpha values ranged from 0.93 to 0.94, and item–total correlation coefficients ranged from 0.65 to 0.81, indicating strong internal consistency. When any single item was removed, the overall Cronbach’s alpha remained stable between 0.93 and 0.94, staying below the 0.95 threshold that may suggest redundancy. Test-retest reliability, assessed in a subsample of 30 participants, yielded an ICC of 0.93 (95% CI, 0.89–0.96), demonstrating excellent temporal stability of the TC-POD-10. These findings support the retention of all 10 items in the final version.

## Discussion

The present study is the first to validate the use of the POD-10 for screening dysphagia in Chinese-speaking patients with oral cancer undergoing facial free-flap reconstruction surgery. The 10-item TC-POD-10 demonstrated strong internal consistency and validity. The ROC analysis revealed that a TC-POD-10 score of ≥ 15 effectively identified dysphagia in this patient population, with sensitivity, specificity, and AUC values of 0.89, 0.96, and 0.92, respectively. Given the adequate sample size and rigorous research methodology, this study’s findings are robust and reliable.

The present study also supports the cross-cultural validity of the TC-POD-10. The rigorous translation and adaptation process ensured semantic, idiomatic, experiential, and conceptual equivalence between the Japanese and Traditional Chinese versions [4, 24, 32]. Minor linguistic modifications were made to enhance cultural relevance for Mandarin-speaking Taiwanese patients, particularly in expressions describing oral discomfort and social participation. For instance, the Japanese phrase “口の中がこぼれる” (“food spills out of the mouth”) was adapted to “吃東西容易從嘴角流出” to convey the same meaning more naturally in Mandarin. Similarly, the item referring to “difficulty eating in front of others” was translated as “不敢在他人面前進食,” reflecting the sociocultural emphasis on modesty and interpersonal harmony in Taiwanese culture. These adjustments helped ensure that the translated items were linguistically clear, culturally resonant, and conceptually consistent with the original instrument. Furthermore, The strong psychometric performance of the TC-POD-10 indicates that the construct of postoperative oral dysfunction is culturally transferable and clinically applicable across different language contexts. Dysphagia was prevalent in the study population, with 52.0% classified as having swallowing difficulties during hospitalization in the plastic surgery ward and an average POD-10 score of 14.6. These findings are consistent with those of previous studies reporting dysphagia rates ranging from 41.3% to 88.0%, reaching 98.0% by postoperative day 7 [1–3].

The present study identified a TC-POD-10 cutoff score of ≥ 15 as the optimal screening threshold for dysphagia risk in patients with oral cancer undergoing free-flap reconstruction surgery, which differs from the cutoff score of 24 reported in the original article [4]. This discrepancy may be attributed to differences in reference standards used between the two studies. Specifically, the present study used the 3-ounce WST combined with dysphagia diagnosis confirmed by both speech therapists and physicians, whereas Matsuda et al. (2021) employed the FOIS and Mini Nutritional Assessment–Short Form, neither of which is considered a gold standard for dysphagia diagnosis [4]. In addition, linguistic and cultural differences may influence patients’ self-perception of swallowing function, resulting in lower self-reported symptom severity in our population. Clinically, adopting a lower cutoff increases sensitivity for early dysphagia detection [24, 32], enabling timely referral to speech therapy and initiation of individualized rehabilitation. Therefore, the cutoff value of ≥ 15 should be interpreted primarily as a screening threshold to identify patients at risk for dysphagia rather than as a definitive diagnostic benchmark. This sensitivity-prioritized approach allows early recognition and timely intervention, while patients with higher scores may subsequently undergo instrumental assessments such as VFSS or FEES for diagnostic confirmation.

In the present study, the TC-POD-10 demonstrated acceptable criterion validity when compared with the overall FOIS score and exhibited significant known-group validity on the basis of the EAT-10 score. These results are consistent with the findings of a previous study that evaluated the original version of the scale, thereby supporting the robustness and consistency of the TC-POD-10 in these populations [4]. However, both the FOIS and EAT-10 were not originally developed for patients with oral cancer undergoing free-flap reconstruction. The FOIS primarily assesses functional oral intake in patients with dysphagia, whereas the EAT-10 serves as a general screening tool for dysphagia symptoms and does not specifically address the unique challenges encountered by this patient group [16, 17]. Despite these limitations, the TC-POD-10 demonstrated satisfactory performance in this study population. Overall, the findings suggest that the TC-POD-10 is a practical, time-efficient, and reliable instrument for evaluating dysphagia in patients with oral cancer who have undergone free-flap reconstruction. Its ease of use and strong psychometric properties make it a valuable tool for both clinical practice and future research in this specific patient population. The TC-POD-10 is a brief, self-administered questionnaire that can be readily integrated into standard postoperative care and rehabilitation workflows. Requiring approximately five minutes to complete, it can be used by clinicians during daily rounds or by rehabilitation teams at follow-up visits. The results can inform early multidisciplinary discussions regarding dietary progression and swallowing therapy, promoting patient-centered care without increasing clinical workload.

A key finding of the present study was the result of the EFA, which accounted for 65.6% of the total variance in the TC-POD-10, indicating a strong underlying factor structure for assessing dysphagia among patients with oral cancer who have undergone free-flap reconstruction. The original POD-10 scale has not been previously subjected to EFA; therefore, the current study provides novel evidence supporting the structural validity of the translated version. Given the absence of EFA in the validation of the original scale, future studies should perform similar analyses on the original POD-10 to determine whether the factor structure observed in the present study is consistent across different language versions and clinical populations. Such investigations would contribute to a more comprehensive understanding of the scale’s construct validity and cross-cultural applicability.

### Limitations

This study has several limitations. First, it was conducted at a single medical center, which may limit the generalizability of the findings. Second, objective dysphagia assessment tools such as the VFSS and FEES were not employed to further validate the TC-POD-10. Finally, although the TC-POD-10 demonstrated strong validity, future studies with larger and more diverse samples are warranted to perform subgroup analyses by tumor site, reconstruction flap type, and prior adjuvant therapy to further elucidate clinical variations in swallowing outcomes.

## Conclusion

The TC-POD-10 demonstrated strong reliability and validity, establishing it as a valuable screening tool for dysphagia in patients undergoing facial free-flap reconstruction surgery for oral cancer. This tool can enhance swallowing health in this specific patient population. Future studies should consider expanding the sample to include more diverse patient groups and further validating the reliability and validity of the POD-10. In addition, objective assessments, such as the VFSS and FEES, can be used to establish correlations between TC-POD-10 results and dysphagia characteristics, thereby strengthening internal validity.

## Data Availability

Data will be made available based on reasonable request.

## References

[1] Klingelhöffer C, Obst A, Ettl T, Meier J, Müller S, Reichert T, Spanier G. Severe postoperative dysphagia as an early predictor for decreased overall survival in patients with oral cancer. J Cranio-Maxillofacial Surg. 2019;47(9):1363–9. 10.1016/j.jcms.2019.06.011. 31331846

[2] Hasegawa T, Yatagai N, Furukawa T, Wakui E, Saito I, Takeda D, Kakei Y, Sakakibara A, Nibu K-i, Akashi M. The prospective evaluation and risk factors of dysphagia after surgery in patients with oral cancer. J Otolaryngology-Head Neck Surg. 2021;50(1):4. 10.1186/s40463-020-00479-6. PMC7830751 33494830

[3] Ohashi Y, Shiga K, Katagiri K, Saito D, Oikawa S-i, Tsuchida K, Ikeda A, Miyaguchi J, Kusaka T, Yamada H. Evaluation and comparison of oral function after resection of cancer of the upper gingiva in patients who underwent reconstruction surgery versus those treated with a prosthesis. BMC Oral Health. 2021;21:1–7. 10.1186/s12903-021-01709-7. PMC8283937 34266443

[4] Matsuda Y, Kumakura I, Okui T, Karino M, Aoi N, Okuma S, Takeda M, Hayashida K, Sakamoto T, Kanno T. Development of a subjective symptom rating scale for postoperative oral dysfunction in patients with oral cancer: reliability and validity of the postoperative oral dysfunction scale-10. Diagnostics. 2021;11(11):2061. 10.3390/diagnostics11112061. PMC8618035 34829408

[5] Monroe D, Pyne JM, McLennan S, Kimmis R, Yoon J, Biron VL. Characteristics and outcomes of transoral robotic surgery with free-flap reconstruction for oropharyngeal cancer: a systematic review. J Robotic Surg. 2023;17(4):1287–97. 10.1007/s11701-023-01572-4. 36964850

[6] Vucak MC, Masic T, Hassouba M, Hatibovic H, Babajic E. Reconstructive option of extensive head and neck defects in cancer surgery. Med Archives. 2013;67(4):275. 10.5455/medarh.2013.67.275-277. 24520753

[7] Cristofaro MG, Barca I, Ferragina F, Novembre D, Ferro Y, Pujia R, Montalcini T. The health risks of dysphagia for patients with head and neck cancer: a multicentre prospective observational study. J Translational Med. 2021;19:1–8. 10.1186/s12967-021-03144-2. PMC8607588 34809654

[8] Frowen J, Hughes R, Skeat J. The prevalence of patient-reported dysphagia and oral complications in cancer patients. Support Care Cancer. 2020;28:1141–50. 10.1007/s00520-019-04921-y. 31203510

[9] Arrese LC, Schieve HJ, Graham JM, Stephens JA, Carrau RL, Plowman EK. Relationship between oral intake, patient perceived swallowing impairment, and objective videofluoroscopic measures of swallowing in patients with head and neck cancer. Head Neck. 2019;41(4):1016–23. 10.1002/hed.25542. 30549151

[10] Logemann JA. Manual for the videofluorographic study of swallowing. No Title); 1993.

[11] Langmore SE, Kenneth SM, Olsen N. Fiberoptic endoscopic examination of swallowing safety: a new procedure. Dysphagia. 1988;2:216–9. 10.1007/BF02414429. 3251697

[12] Dysphagia UEA. European Society for Swallowing Disorders. Dysphagia. 2013;28:280–335. 10.1007/s00455-013-9455-z.

[13] Schindler A, Denaro N, Russi EG, Pizzorni N, Bossi P, Merlotti A, Bissetti MS, Numico G, Gava A, Orlandi E. Dysphagia in head and neck cancer patients treated with radiotherapy and systemic therapies: literature review and consensus. Crit Rev Oncol/Hematol. 2015;96(2):372–84. 10.1016/j.critrevonc.2015.06.005. 26141260

[14] Zhang Y, Wang Y, Zhu Y, Wan H. Diagnostic value of water swallow test for dysphagia in patients with head and neck cancer: A systematic review and meta-analysis. Head Neck. 2024;46(5):1210–23. 10.1002/hed.27713. 38445384

[15] Chen AY, Frankowski R, Bishop-Leone J, Hebert T, Leyk S, Lewin J, Goepfert H. The development and validation of a dysphagia-specific quality-of-life questionnaire for patients with head and neck cancer: the MD Anderson dysphagia inventory. Archives Otolaryngology–Head Neck Surg. 2001;127(7):870–6. 10.1001/pubs.ArchOtolaryngol. 11448365

[16] Belafsky PC, Mouadeb DA, Rees CJ, Pryor JC, Postma GN, Allen J, Leonard RJ. Validity and reliability of the Eating Assessment Tool (EAT-10). Annals of Otology. Rhinology Laryngology. 2008;117(12):919–24. 10.1177/000348940811701210. 19140539

[17] Crary MA, Mann GDC, Groher ME. Initial psychometric assessment of a functional oral intake scale for dysphagia in stroke patients. Arch Phys Med Rehabil. 2005;86(8):1516–20. 10.1016/j.apmr.2004.11.049. 16084801

[18] Huang PS. (2023). Reliability and validity of the Chinese version of the Eating Assessment Tool-10 https://hdl.handle.net/11296/zsf596. (Master’s thesis). Department of Special Education, Speech-Language Pathology Program, University of Taipei.

[19] Bachmann AS, Höche S, Peters B, Wiltfang J, Hertrampf K. Effects of high-frequency speech therapy on speech-related quality of life and objective speech intelligibility of oral cancer patients. J Cranio-Maxillofacial Surg. 2021;49(11):1072–80. 10.1016/j.jcms.2021.06.011. 34193379

[20] Barclay C, Foster E, Taylor C. Restorative aspects of oral cancer reconstruction. Br Dent J. 2018;225(9):848–54. 10.1038/sj.bdj.2018.927. 30412540

[21] Lazarus C, Logemann JA, Pauloski BR, Rademaker AW, Helenowski IB, Vonesh EF, MacCracken E, Mittal BB, Vokes EE, Haraf DJ. Effects of radiotherapy with or without chemotherapy on tongue strength and swallowing in patients with oral cancer. Head Neck: J Sci Specialties Head Neck. 2007;29(7):632–7. 10.1002/hed.20577. 17230558

[22] Li W-T, Hsieh J-H, Horng S-Y, Cheng N-C, Chien H-F, Chen J-S, Lai H-S. Treatment and long-term follow-up of oral cancer postoperative sialorrhea with dermal sling operation. Ann Plast Surg. 2015;74:S113–7. 10.1097/SAP.0000000000000466. 25774969

[23] Alsubaie HM, Sayed SI, Alsini AY, Alkaff HH, Margalani OA, Abu-Zaid A, Abu-Suliman OA, Alherabi AZ, Alghamdi SA, Saleh E. Validity and reliability of an Arabic version of MD Anderson dysphagia inventory (MDADI). Dysphagia. 2022;37(4):946–53. 10.1007/s00455-021-10356-7. 34427776

[24] Beaton DE, Bombardier C, Guillemin F, Ferraz MB. Guidelines for the process of cross-cultural adaptation of self-report measures. Spine. 2000;25(24):3186–91. 10.1097/00007632-200012150-00014 11124735

[25] Steiner DL, Norman GR. Health Measurement Scales: A Practical Guide to Thier Development and Use. Oxford University Press; 1995.

[26] Streiner DL, Norman GR, Cairney J. Health measurement scales: a practical guide to their development and use. Oxford University Press; 2024.

[27] Zhou H, Zhu Y, Zhang X. Validation of the Chinese version of the functional oral intake scale (FOIS) score in the assessment of acute stroke patients with dysphagia. In MEDINFO 2017: Precision Healthcare through Informatics. 2017:1195–1199. IOS Press. 10.3233/978-1-61499-830-3-1195. 29295292

[28] Ke WC. (2020). Reliability and validity analysis of the Taiwan version of the Functional Oral Intake Scale: A study on head and neck cancer patients https://hdl.handle.net/11296/3ap9wb. [Unpublished master’s thesis]. University of Taipei, Department of Special Education, Speech-Language Pathology Program.

[29] Suiter DM, Leder SB, Karas DE. The 3-ounce (90-cc) water swallow challenge: a screening test for children with suspected oropharyngeal dysphagia. Otolaryngology-Head Neck Surg. 2009;140(2):187–90. 10.1016/j.otohns.2008.11.016. 19201286

[30] Mandrekar JN. Receiver operating characteristic curve in diagnostic test assessment. J Thorac Oncol. 2010;5(9):1315–6. 10.1097/JTO.0b013e3181ec173d. 20736804

[31] Bewick V, Cheek L, Ball J. Statistics review 13: receiver operating characteristic curves. Crit Care. 2004;8:1–5. 10.1186/cc3000. PMC1065080 15566624

[32] Swami V, Barron D. Translation and validation of body image instruments: Challenges, good practice guidelines, and reporting recommendations for test adaptation. Body image. 2019;31:204–20. 10.1016/j.bodyim.2018.08.014. 30220631

